# Editorial: The Role of Mobile Genetic Elements in Bacterial Evolution and Their Adaptability

**DOI:** 10.3389/fmicb.2022.849667

**Published:** 2022-02-21

**Authors:** Filipa F. Vale, Philippe Lehours, Yoshio Yamaoka

**Affiliations:** ^1^Pathogen Genome Bioinformatics and Computational Biology, Research Institute for Medicines (iMed-ULisboa), Faculty of Pharmacy, Universidade de Lisboa, Lisboa, Portugal; ^2^INSERM U1312, Bordeaux Institute of Oncology, BRIC, Bordeaux, France; ^3^Centre National de Référence des Campylobacters et des Hélicobacters, Bordeaux, France; ^4^Department of Environmental and Preventive Medicine, Faculty of Medicine, Oita University, Oita, Japan

**Keywords:** mobile elements (MEs), evolution, adaptability, bacteriophage, virulence factors, insertion sequence (Is), restriction and modification

The bacterial mobilome, i.e., the group of all mobile elements present in a bacterial genome, are important players in bacterial evolution, shaping the host genome through complex interactions between the mobile element and host bacteria. Mobile element is a generic term for a multitude of genomic sequences, such as plasmids, prophages, pathogenicity islands, restriction and modification systems, transposons, and insertion sequences, among others, that share the ability to be transmitted vertically with cell division or through horizontal transfer. Thus, they are able to move within the host genome as well as jump across genomes, shaping and co-evolving with chromosomal genomes. Mobile elements can change their insertion location, copy number, provide new gene functions, or affect chromosomal gene expression. Mobile elements are known to potentiate gene gain and loss, a major force that can profoundly change bacterial fitness. This change can contribute to the genetic adaptation to new environments and the emergence of divergent bacterial populations that may produce evolutionary distinct species.

Since their discovery, the mechanisms of persistence and interaction of mobile elements with the bacterial genome have been increasingly studied. This special issue, through the presentation of pertinent and important examples of distinct mobile elements (genomic islands, pathogenicity islands, insertion sequences, prophages, and restriction and modifications systems), either by reviewing, showing new aspects, or presenting new bioinformatics tools, shows the importance of comprehending the biology of mobile genetic elements.

To start, the role of distinct mobile genetic elements in adaptive evolution is addressed.

The epigenetic DNA-based methylation plays important roles in gene expression regulation. Yano et al. described a gene expression regulation network in *H. pylori* consisting of many DNA methyltransferases each frequently changing its target sequence-specificity in search for adaptation. *H. pylori* carries a large and variable repertoire of sequence-specific DNA methyltransferases, and motility, oxidative stress tolerance, and DNA damage repair are likewise regulated by multiple methyltransferases. The network provides a new paradigm in understanding global gene regulation and adaptive evolution.

Moving into another microorganism, *Escherichia coli* presents many accessory genes acquired by horizontal transfer that form syntenic blocks and are recognized as genomic islands (GIs). These genomic regions contribute to the rapid evolution, diversification, and adaptation of *E. coli* variants. In the paper by Desvaux et al. the authors reviewed a subgroup of GIs from *E. coli* termed pathogenicity islands which contribute to the emergence of virulent bacteria and to the development of intestinal and extraintestinal diseases.

Next, a group of manuscripts highlights the role of mobile genetic elements in bacteria pathogenicity, or their use for epidemiologic purposes.

Fischer et al. focused again on the pathogenic bacterium *H. pylori*, which was acquired multiple type IV secretion systems (T4SSs). *H. pylori* encodes up to four T4SSs on its chromosome, namely the Cag T4SS present in the *cag* pathogenicity island (*cag*PAI), the ComB system, as well as the Tfs3 and Tfs4 T4SSs, some of which exhibit unique T4SS functions. The authors nicely highlighted the current knowledge on these four T4SSs and the involved mechanisms with consequences for *H. pylori* adaptation to the hostile environment in the human stomach.

Insertions sequences (IS), in particular IS900, is exclusively present in *Mycobacterium avium* subsp. *paratuberculosis* (Map), being used as an epidemiological marker. Conde et al. developed a bioinformatics tool, named IS900 RFLP in silico, that analyses the distribution and polymorphisms of IS900 by performing an in silico RFLP analysis, over complete Map genomes. Thus, the developed tool contributes to the better understanding of Map evolution and to studying the biological implications of IS900 insertions.

Radiation-resistant bacterium *Deinococcus geothermalis* has a total of 73 IS in genomes, and some of them are actively transposed to other loci with replicative mode due to the oxidative stress of hydrogen peroxide treatment. Lee et al. described that LysR family member gene disrupted strain (Δ*dgeo*_2840) revealed higher resistance to oxidative stress than wild type strain at the late exponential growth phase. The authors concluded that there is a LysR family DNA-binding protein dependent active transposition of specific types of IS and the up-regulated OxyR has not positively controlled ROS scavenger enzymes in *D. geothermalis*.

Last but not least, the mechanism of adaptive immune systems against bacteriophages and the role of prophages in bacterial diversity are addressed.

Diverse CRISPR-Cas systems constitute an indispensable part of the bacterial adaptive immune system against viral infections. However, to escape from this immune system, bacteriophages have also evolved corresponding anti-defense measures. Wang et al. investigated the diversity of CRISPR-Cas systems and the presence of prophages in the genomes of 66 *Bifidobacterium pseudocatenulatum* strains. Their results indicated that the competition between *B. pseudocatenulatum* and phages is a major driving factor for the genomic diversity of both partners.

Sequencing technology is evolving rapidly, and so-called third-generation sequencing technology (such as PacBio RS II) becomes popular. Using PacBio RS II, Xu et al. focused on the *Bacillus velezensis* HAB-2, that is used as a biological control agent in agricultural fields. Comparative analysis of *B. velezensis* HAB-2 with other *Bacillus* spp. strains revealed obvious differences in the prophage region. The prophage regions and genes encoding PPTases may provide novel insight for the bacteriostatic mechanism of *Bacillus* in the biological control of plant diseases.

Bacteriophages are the most common biological entity, establishing complex phage-bacteria interactions. Munoz et al. presented an up-to-date review of the bacteriophages found in the human gut pathogen *H. pylori*. The authors discuss the impact of the prophages in the genomic diversity, virulence, and fitness of *H. pylori*. Moreover, in this study it is examined whether these viral particles can be used as a therapeutic alternative (phage therapy) to address bacterial resistance and improve infection eradication rates.

Taken together, the set of papers offered by this special issue showed that the bacterial mobilome is a key element to address the evolutionary, ecological, and pathogenicity success of bacteria.

## Author Contributions

All authors listed have made a substantial, direct, and intellectual contribution to the work and approved it for publication.

## Conflict of Interest

The authors declare that the research was conducted in the absence of any commercial or financial relationships that could be construed as a potential conflict of interest.

## Publisher's Note

All claims expressed in this article are solely those of the authors and do not necessarily represent those of their affiliated organizations, or those of the publisher, the editors and the reviewers. Any product that may be evaluated in this article, or claim that may be made by its manufacturer, is not guaranteed or endorsed by the publisher.

